# The thicker the endometrium, the better the neonatal outcomes?

**DOI:** 10.1093/hropen/hoad028

**Published:** 2023-07-13

**Authors:** Jing Wu, Jianlei Huang, Jie Dong, Xifeng Xiao, Mao Li, Xiaohong Wang

**Affiliations:** Department of Obstetrics and Gynecology, Reproductive Medicine Center, Tang Du Hospital, The Air Force Military Medical University, Xi’an, China; Department of Obstetrics and Gynecology, Reproductive Medicine Center, Tang Du Hospital, The Air Force Military Medical University, Xi’an, China; Department of Obstetrics and Gynecology, Reproductive Medicine Center, Tang Du Hospital, The Air Force Military Medical University, Xi’an, China; Department of Obstetrics and Gynecology, Reproductive Medicine Center, Tang Du Hospital, The Air Force Military Medical University, Xi’an, China; Department of Obstetrics and Gynecology, Reproductive Medicine Center, Tang Du Hospital, The Air Force Military Medical University, Xi’an, China; Department of Obstetrics and Gynecology, Reproductive Medicine Center, Tang Du Hospital, The Air Force Military Medical University, Xi’an, China

**Keywords:** endometrial thickness (EMT), hCG trigger day, preterm delivery (PTD), gestational age (GA), fresh embryo transfer

## Abstract

**STUDY QUESTION:**

Is endometrial thickness (EMT) on the hCG trigger day related to the neonatal outcome of a single birth after fresh embryo transfer (ET)?

**SUMMARY ANSWER:**

An EMT ≤7.8 mm was an independent predictor for greater odds of preterm delivery (PTD) of singletons born after fresh ET.

**WHAT IS KNOWN ALREADY:**

There may be a positive association between live birth rates and EMT after fresh ET. It is still unknown whether a similar association is seen for the neonatal outcomes of singletons in fresh cycles.

**STUDY DESIGN, SIZE, DURATION:**

This retrospective study involved singleton live births in women undergoing autologous IVF cycles during the period from 1 October 2016 to 31 July 2021.

**PARTICIPANTS/MATERIALS, SETTING, METHODS:**

A total of 2010 women who fulfilled the inclusion criteria were included. A multivariable regression analysis was performed to detect the relationship between EMT and neonatal outcomes after controlling for potential confounders. Smooth curve fitting and threshold effect analysis were used to evaluate the accurate cutoff value of EMT.

**MAIN RESULTS AND THE ROLE OF CHANCE:**

The results of the multivariate regression analyses showed that the odds of PTD were reduced by 45% with an EMT of 9.00–9.90 mm (adjusted odds ratio (OR): 0.55, 95% CI: 0.13 to 0.98; *P* = 0.0451), reduced by 58% with an EMT of 10.00–10.90 mm (adjusted OR: 0.42, 95% CI: 0.06 to 0.87; *P* = 0.0211) and reduced by 75% with an EMT >11 mm (adjusted OR: 0.25, 95% CI: 0.04 to 0.66; *P* = 0.0034), compared to the group with an EMT of 6.00–8.90 mm. It could also be seen from the adjusted smooth curves that the odds of PTD decreased and gestational age (GA) increased with increasing EMT. Combined with the analysis of threshold effects, the results indicated that when the EMT was ≤7.6 mm, the incidence of PTD decreased as the EMT gradually increased (adjusted OR: 0.47, 95% CI: 0.03 to 0.99; *P* = 0.0107), and when the EMT was ≤7.8 mm, the GA increased (adjusted β: 1.94, 95% CI: 1.26 to 2.63; *P* < 0.0001) as the EMT gradually increased.

**LIMITATIONS, REASONS FOR CAUTION:**

The main limitation of our study is its retrospective design. Although we found a significant decrease in PTD as the EMT increased, in terms of GA, the magnitude of the differences was modest, which may limit the clinical relevance of the findings.

**WIDER IMPLICATIONS OF THE FINDINGS:**

Our data provide new insight into the relationship between EMT and neonatal outcomes by indicating that a thin endometrium of ≤7.8 mm is associated with an increased odds of PTD of singletons after fresh ET.

**STUDY FUNDING/COMPETING INTEREST(S):**

This study was supported by the National Natural Science Foundation of China (grant no. 82071717). There are no conflicts of interest.

WHAT DOES THIS MEAN FOR PATIENTS? Endometrial thickness, monitored by a technique called transvaginal ultrasound, is a convenient and commonly used indicator closely related to pregnancy rate during IVF cycles. Previous studies showed a positive association between live birth rates and endometrial thickness after fresh embryo transfers. Is there a similar association for neonatal outcomes, such as birthweight and gestational age?

This analysis of 2010 women under 42 years old who became pregnant following fresh embryo transfers and gave birth to a single live baby between 1 October 2016 and 31 July 2021 at the Center of Assisted Reproduction at Tangdu Hospital of Air Force Military Medical University in China demonstrated that a thin endometrium of ≤7.8 mm was associated with an increased odds of preterm delivery of singletons. This information may be useful for patients to evaluate the risk of preterm birth based on endometrial thickness during fresh IVF treatments.

## Introduction

*In vitro* fertilization has developed rapidly in more than 40 years since the first birth in 1978. Currently, the assessment of the success of IVF is not limited to a satisfactory live birth rate but also relates to fetal safety during pregnancy and the perinatal period. It is well known that singleton pregnancies after fresh IVF cycles have a higher risk of obstetric and perinatal complications, such as low birth weight (LBW), small for gestational age (SGA), placenta previa, and preeclampsia, compared with naturally born infants (Pinborg *et al.*, 2013; Dunietz *et al.*, 2015; Maheshwari *et al.*, 2018). Some studies have shown that these risks are the result of intrinsic factors in subfertile couples or ART itself, including ovarian stimulation, *in vitro* embryo culture, and cryopreservation techniques (Pal *et al.*, 2008; Vergouw *et al.*, 2012; Sunkara *et al.*, 2015). However, it is difficult to know which step mainly leads to adverse effects.

Endometrial thickness (EMT), which is closely related to pregnancy outcomes, is a convenient and commonly used indicator from transvaginal ultrasound monitoring during ovarian stimulation. Many studies have demonstrated that patients with a thin endometrium (<8 mm) have lower chances of becoming pregnant, whether in fresh embryo transfer (ET) cycles or in frozen-thawed ET cycles (Liu *et al.*, 2018; Eftekhar *et al.*, 2019; Shalom-Paz *et al.*, 2021; Zhao *et al.*, 2022), whereas researchers in other studies have claimed that women with a thick endometrium (>14 mm) have lower implantation and pregnancy rates and a higher rate of miscarriage (Weissman *et al.*, 1999).

However, by searching the literature, we found that only a few studies reported the impact of EMT on neonatal outcomes. In singletons who underwent fresh ETs, Chung *et al.* (2006) reported, for the first time, that the risk of LBW in pregnant women with an EMT ≤10 mm was twice as high as that in women with an EMT >12 mm. Another study showed that an EMT <7.5 mm was related to increased risks of SGA babies resulting from fresh ET, while the incidence of LBW did not change (Oron *et al.*, 2018). For maternal complications during the pregnancy period, Liu *et al.* (2021) observed that a thin endometrium (≤8 mm) was associated with an increased risk of hypertensive disorders of pregnancy (HDP). A more recent meta-analysis showed that an EMT <7.5 mm increased the incidence of both HDP and SGA infants and decreased the birthweight of babies (Liao *et al.*, 2021). However, Huang *et al.* (2020) showed that EMT was not independently associated with adverse neonatal outcomes. The influence of EMT on neonatal outcomes is currently not fully understood. All previous studies were designed with simply three or four groups. Therefore, if a thin EMT is harmful to neonatal health, the exact cutoff value of the EMT is still unclear.

The aim of our study was to investigate the relationship between EMT on the hCG trigger day and neonatal outcomes of singletons after fresh ET. In addition, we tried to explore the exact cutoff value of the EMT for affected outcomes. To our knowledge, this is the first study in which smooth curve fitting and threshold effect analysis have been used to evaluate the accurate cutoff value of EMT.

## Materials and methods

### Study population

**Figure 1.**
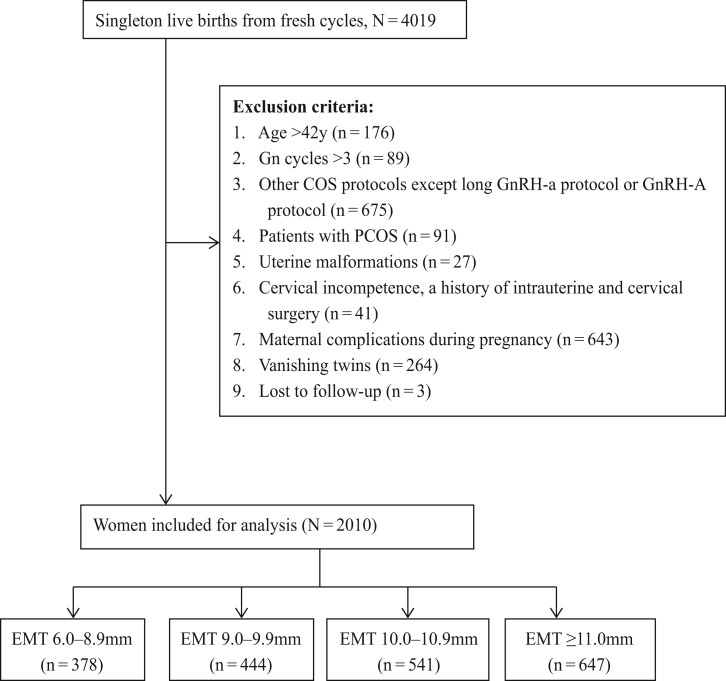
**Flowchart of patient inclusion.** Gn: gonadotrophin; COS: (controlled) ovarian stimulation; GnRH-a: GNRH agonist; GnRH-A: GnRH antagonist; PCOS: polycystic ovary syndrome; EMT: endometrial thickness.

We conducted a retrospective cohort study covering the interval between 1 October 2016 and 31 July 2021, at the Center of Assisted Reproduction at Tangdu Hospital of Air Force Military Medical University in China. Our study population included all women who met the following criteria: autologous IVF/ICSI cycles, long luteal GnRH agonist (GnRH-a) protocol or antagonist protocol during the ovarian stimulation process, age at oocyte retrieval ≤42 years, and live singleton birth after fresh ET. All patients were in their first three gonadotrophin cycles. In this study, patients with known polycystic ovary syndrome (PCOS) diagnosed by the Rotterdam criteria or suffering from maternal complications during pregnancy were excluded from the analysis. This decision was made to prevent any basis for neonatal outcomes. In addition, patients with multiple births, vanishing twins, uterine malformations, cervical incompetence, or a history of intrauterine and cervical surgery were also excluded. A flow diagram of the patient-selection process is shown in Fig. 1. Finally, a total of 2010 cycles were included in this study.

### Ethics approval

This study was approved by the Institutional Review Board of the hospital (assigned number: TDLL-202203-02).

### Study procedures

Ovarian stimulation was achieved by using the GnRH agonist long protocol or the GnRH antagonist protocol. The starting dose and the type of ovarian stimulation protocol were determined by the patients’ characteristics and clinician preferences. All patients received both recombinant and urinary exogenous gonadotrophins. The daily gonadotrophins dose was decided on follicular growth in successive transvaginal sonograms and a blood test that included evaluation of the plasma levels of estrogen (E2), progesterone, and LH until the day of the hCG trigger. Ovulation was triggered in all patients with 250 μg of recombinant hCG when there were at least three follicles ≥17-mm diameter on transvaginal ultrasound. Ultrasound-guided oocyte retrieval was performed 36 h after the trigger injection. Approximately 12–17 h after insemination or sperm injection, the oocytes were examined for fertilization. Cleavage-stage embryos were transferred on the third day and blastocysts were transferred on the fifth day after oocyte retrieval (Pereira *et al.*, 2015). There were no major changes in the clinical and laboratory conditions, culture media, or fresh ET techniques during the study period.

### Outcome measures and definitions

Data on demographic and cycle characteristics were collected, including the ages of the couples, maternal BMI, anti-Müllerian hormone (AMH) levels, basal FSH levels, type of infertility, prior gonadotrophin cycles, infertility duration, infertility cause, total gonadotrophin dose, stimulation duration, estrogen (E2) levels on the trigger day, number of oocytes retrieved, stages and numbers of transferred embryos, and the thickness and type of endometrium.

The newborn height, weight, gender, gestational age (GA), and mode of delivery were recorded for all live infants. GA was counted from the day of ET, which was identified as Day 17 of the cycle for cleavage-stage ET and Day 19 for blastocyst transfer (Nelissen *et al.*, 2012). Preterm delivery (PTD) and very PTD were identified as GA 32–36 weeks and <32 weeks, respectively. LBW, very LBW, and fetal macrosomia were identified as birthweight <2500, <1500, and ≥4000 g, respectively. SGA and very SGA were identified as birthweight <10th and <3rd percentiles. Large for gestational age (LGA) and very LGA were identified as birthweights >90th and >97th percentiles, respectively. In addition, the Z-score was introduced to calculate birthweight accounting for GA and neonatal sex according to the following equation: Z-score = (*x* − *μ*)/*σ*, in which *x* is the weight of newborns, and *μ* and *σ* are the average birthweight and the SD of infants of the same sex and the same GA, respectively. Birthweight percentiles and the calculation of Z-score were based on Chinese reference singleton newborns stratified by GA and neonatal sex (Dai *et al.*, 2014).

### Statistical analysis

Categorical variables are expressed as the number of cases (n) with the percentage of occurrence (%), and continuous variables are expressed as the median (interquartile range) or mean ± SD as appropriate. The patient demographics, cycle characteristics, and neonatal outcomes were compared between the four groups (according to EMT) via *t* tests (for continuous variables) or *χ*^2^ tests (for categorical variables).

Multiple linear regression analysis was performed to explore the relationship between EMT (mm) and GA (weeks). Logistic regression analysis was used to assess the adverse categorical outcomes, such as LBW, very LBW, fetal macrosomia, preterm, very preterm, SGA, very SGA, LGA, and very LGA, with adjustments for potential confounding factors.

We selected the confounders on the basis of their associations with the outcomes of interest or a change in the effect estimate of more than 10%, including the ages of the couples, maternal BMI, basal FSH levels, AMH levels, type of infertility, prior gonadotropin cycle, ovarian stimulation protocols, dosage of gonadotropins, E2 level on the hCG trigger day, number of oocytes retrieved, fertilization method, number and stage of embryos transferred, and gender of newborn.

All statistical analyses were performed by using EmpowerStats (www.empowerstats.com, X&Y Solutions, Inc., Boston, MA, USA) and R software version 3.6.1 (http://www.r-project.org). A *P*-value of <0.05 was considered to be statistically significant.

## Results

A total of 2010 fresh IVF cycles that met the inclusion criteria during the study period were included in this analysis. Of the births, 378, 444, 541, and 647 live-born singletons were categorized as Group 1 (6.00–8.90 mm), Group 2 (9.00–9.90 mm), Group 3 (10.00–10.90 mm), and Group 4 (>11.00 mm), respectively.

The patient baseline characteristics, cycle parameters, and neonatal outcomes are presented in Table 1. Compared to Group 1, women in the thicker endometrium groups were younger and had lower basal FSH levels, higher AMH values, and more primary infertility. Furthermore, in terms of the cycle parameters, the thicker endometrium groups had more GnRH agonist long protocols, higher E2 levels on the hCG trigger day, more total retrieved oocytes, lower dosages of gonadotropins, more ICSI cycles, and fewer cleavage embryos transferred.

**Table 1:** Patient baseline characteristics, cycle parameters, and neonatal outcomes by different groups of endometrial thickness on the hCG trigger day.

Characteristics	6.00–8.90 mm	9.00–9.90 mm	10.00–10.90 mm	>11.00 mm	*P*-value
	(n = 378)	(n = 444)	(n = 541)	(n = 647)	
**Baseline characteristics**					
Maternal age (year), mean ± SD	31.50 ± 4.32	30.78 ± 3.90	30.64 ± 3.88	30.58 ± 3.91	0.015*
Paternal age (year), mean ± SD	33.00 ± 5.01	32.52 ± 4.85	32.43 ± 4.73	32.09 ± 4.59	0.105
Maternal BMI (kg/m^2^), mean ± SD	22.22 ± 2.94	22.22 ± 3.10	22.07 ± 2.96	22.60 ± 3.42	0.103
Basal FSH (IU/l), mean ± SD	7.83 ± 2.46	7.91 ± 2.33	7.66 ± 2.51	7.35 ± 2.44	<0.001*
AMH (ng/ml), mean ± SD	2.85 ± 2.34	2.64 ± 2.06	2.81 ± 2.20	3.03 ± 2.34	0.035*
Type of infertility, n (%)					<0.001*
Primary	146 (38.62%)	220 (49.55%)	282 (52.13%)	371 (57.34%)	
Secondary	232 (61.38%)	224 (50.45%)	259 (47.87%)	276 (42.66%)	
Infertility duration (year), mean ± SD	3.61 ± 2.96	3.59 ± 2.71	3.77 ± 2.70	3.74 ± 2.57	0.660
Infertility cause, n (%)					0.234
Female	259 (68.52%)	292 (65.77%)	360 (66.54%)	393 (60.74%)	
Male	68 (17.99%)	88 (19.82%)	93 (17.19%)	148 (22.87%)	
Mixed	33 (8.73%)	38 (8.56%)	57 (10.54%)	61 (9.43%)	
Unexplained	18 (4.76%)	26 (5.86%)	31 (5.73%)	45 (6.96%)	
Prior gonadotropin cycle, mean ± SD	1.24 ± 0.65	1.24 ± 0.57	1.23 ± 0.53	1.20 ± 0.50	0.536
**Cycle parameters**					
Ovarian stimulation protocols, n (%)					0.025*
GnRH-a long protocol	230 (60.85%)	268 (60.36%)	343 (63.40%)	434 (67.08%)	
Antagonist protocol	148 (39.15%)	176 (39.64%)	198 (36.60%)	213 (32.92%)	
Dosage of gonadotropins (IU), mean ± SD	2309.84 ± 1111.45	2375.34 ± 1086.39	2307.30 ± 1150.48	2202.80 ± 1109.18	0.017*
Stimulation duration (days), mean ± SD	11.40 ± 2.35	11.54 ± 2.11	11.62 ± 2.18	11.66 ± 2.18	0.324
E2 level on HCG day (pg/ml), mean ± SD	2809.38 ± 1299.41	2969.08 ± 1255.00	3089.74 ± 1316.63	3051.18 ± 1215.43	0.006*
Number of oocytes retrieved, mean ± SD	9.17 ± 3.87	9.72 ± 3.90	9.62 ± 3.95	9.95 ± 3.64	0.018*
Retrieved MII oocytes, mean ± SD	7.97 ± 3.40	8.27 ± 3.57	8.28 ± 3.60	8.54 ± 3.32	0.089
Fertilization method, n (%)					0.023*
IVF	273 (72.22%)	292 (65.77%)	363 (67.10%)	403 (62.29%)	
ICSI	93 (24.60%)	129 (29.05%)	146 (26.99%)	212 (32.77%)	
IVF + ICSI	12 (3.17%)	23 (5.18%)	32 (5.91%)	32 (4.95%)	
Fertilization rate (%), mean ± SD	83.58 ± 15.56	81.84 ± 16.92	83.07 ± 16.55	83.58 ± 16.07	0.320
Number of available embryos, mean ± SD	4.27 ± 2.30	4.55 ± 2.47	4.42 ± 2.33	4.70 ± 2.33	0.071
Stage embryo transferred, n (%)					0.001*
D3	332 (87.83%)	376 (84.68%)	455 (84.10%)	510 (78.83%)	
D5	46 (12.17%)	68 (15.32%)	86 (15.90%)	137 (21.17%)	
Number of embryos transferred, n (%)					0.188
1	95 (25.13%)	116 (26.13%)	139 (25.69%)	200 (30.91%)	
2	282 (74.60%)	328 (73.87%)	401 (74.12%)	444 (68.62%)	
3	1 (0.26%)	0 (0.00%)	1 (0.18%)	3 (0.46%)	
Endometrial thickness (mm), mean ± SD	7.84 ± 0.68	9.11 ± 0.23	10.08 ± 0.20	11.70 ± 0.90	<0.001*
Endometrial type, n (%)					0.296
A	7 (1.85%)	11 (2.48%)	7 (1.29%)	5 (0.77%)	
A-B	20 (5.29%)	24 (5.41%)	34 (6.28%)	29 (4.48%)	
B	120 (31.75%)	142 (31.98%)	154 (28.47%)	204 (31.53%)	
B-C	205 (54.23%)	234 (52.70%)	316 (58.41%)	354 (54.71%)	
C	26 (6.88%)	33 (7.43%)	30 (5.55%)	55 (8.50%)	
**Neonatal outcomes indicators**					
Gender of newborn, n (%)					0.507
Male	203 (53.70%)	215 (48.42%)	277 (51.20%)	327 (50.54%)	
Female	175 (46.30%)	229 (51.58%)	264 (48.80%)	320 (49.46%)	
Newborn height (cm), mean ± SD	50.22 ± 2.23	50.22 ± 1.99	50.14 ± 1.98	50.19 ± 2.29	0.681
GA (week), mean ± SD	38.74 ± 2.00	38.96 ± 1.68	39.06 ± 1.52	39.08 ± 1.73	0.013*
Birthweight (g), mean ± SD	3123.48 ± 491.04	3201.79 ± 556.65	3191.90 ± 446.38	3276.62 ± 495.47	0.025*
Z-score, mean ± SD	0.05 ± 1.15	0.07 ± 1.13	−0.05 ± 1.00	0.19 ± 1.02	0.220
Birthweight, n (%)					0.031*
Normal birthweight	342 (90.48%)	373 (84.01%)	494 (91.31%)	574 (88.72%)	
Very low birth weight (<1500 g)	6 (1.59%)	5 (1.13%)	0 (0.00%)	6 (0.93%)	
Low birthweight (<2500 g)	26 (6.88%)	44 (9.91%)	16 (2.96%)	23 (3.55%)	
Fetal macrosomia (≥4000 g)	4 (1.05%)	22 (4.95%)	31 (5.73%)	44 (6.80%)	
Gestational age, n (%)					0.023*
Full-term (≥37 weeks)	319 (84.39%)	400 (90.09%)	500 (92.42%)	614 (94.90%)	
Preterm (32–36 weeks)	48 (12.70%)	39 (8.78%)	41 (7.58%)	24 (3.71%)	
Very preterm (<32 weeks)	11 (2.91%)	5 (1.13%)	0 (0.00%)	9 (1.39%)	
Small for gestational age, n (%)	26 (6.88%)	37 (8.33%)	43 (7.95%)	43 (6.65%)	0.686
Very small for gestational age, n (%)	5 (1.32%)	11 (2.48%)	14 (2.59%)	10 (1.55%)	0.384
Large for gestational age, n (%)	40 (10.58%)	39 (8.78%)	53 (9.80%)	51 (7.88%)	0.469
Very large for gestational age, n (%)	16 (4.23%)	13 (2.93%)	19 (3.51%)	24 (3.71%)	0.789
Mode of delivery, n (%)					0.155
Vaginal	121 (32.01%)	154 (34.68%)	183 (33.83%)	249 (38.49%)	
Caesarean section	257 (67.99%)	290 (65.32%)	358 (66.17%)	398 (61.51%)	

AMH: anti-Müllerian hormone; E2: estrogen; GA: gestational age; GnRH-a: GNRH agonist.

* Statistically significant, with *P *<* *0.05.

In terms of neonatal outcomes, with increasing EMT on the hCG trigger day, both the GA (weeks) and birthweight (g) gradually increased, and the proportion of LBW infants and the incidence of PTD babies decreased. The differences in all of the above-mentioned indices were significant (*P* < 0.05).

The neonatal outcomes of the multivariate analyses are shown in Table 2. GA (weeks) was positively associated with increasing EMT on the hCG trigger day (adjusted β: 0.13, 95% CI: 0.03 to 0.29; *P* < 0.001), even after accounting for confounding variables. The effective value β implied that for every 1 mm increase in EMT, GA increased by 0.13 weeks. Moreover, when EMT was taken as the categorical indicator, GA (weeks) still exhibited an increasing trend with the increase in EMT in the four groups (38.60 ± 1.99 vs 38.91 ± 1.68 vs 39.06 ± 1.52 vs 39.17 ± 1.73, *P* trend <0.0001). When the EMT was 9.0–9.9 mm (adjusted β: 0.29, 95% CI: 0.03 to 0.48; *P* = 0.0226), 10.0–10.9 mm (adjusted β: 0.47, 95% CI: 0.15 to 0.69; *P* = 0.0019) and >11 mm (adjusted β: 0.55, 95% CI: 0.20 to 0.83; *P* = 0.0005), the GA increase was significant compared to the 6.00–8.90 mm group.

**Table 2:** Multivariable regression analysis for neonatal outcomes by endometrial thickness.

Characteristics	Gestational age (weeks)	Non-adjusted β/OR	*P*-value	Adjusted β/OR	*P*-value
	Adjust mean (95% CI)	(95% CI)		(95% CI)	
Gestational age (week)		0.07 (0.02, 0.12)	0.0067	0.13 (0.03, 0.29)	<0.0001*
Gestational age (EMT categorized into four groups)					
6.00–8.90 mm (n = 378)	38.60 ± 1.99	Ref		Ref	
9.00–9.90 mm (n = 444)	38.91 ± 1.68	0.22 (−0.01, 0.46)	0.0659	0.29 (0.03, 0.48)	0.0226*
10.00–10.90 mm (n = 541)	39.06 ± 1.52	0.32 (0.09, 0.55)	0.0055	0.47 (0.15, 0.69)	0.0019*
>11.00 mm (n = 647)	39.17 ± 1.73	0.34 (0.12, 0.56)	0.0021	0.55 (0.20, 0.83)	0.0005*
*P* trend	<0.0001*				
Preterm (32–36 weeks)		0.77 (0.63, 0.95)	0.0129	0.55 (0.20, 0.75)	0.0062*
Preterm (EMT categorized into four groups)					
6.00–8.90 mm (n = 378)		Ref		Ref	
9.00–9.90 mm (n = 444)		0.66 (0.31, 1.41)	0.2865	0.55 (0.13, 0.98)	0.0451*
10.00–10.90 mm (n = 541)		0.57 (0.26, 1.23)	0.1512	0.42 (0.06, 0.87)	0.0211*
>11.00 mm (n = 647)		0.26 (0.10, 0.65)	0.0041	0.25 (0.04, 0.66)	0.0034*
Very preterm (<32 weeks)		0.10 (0.01, 0.78)	0.0287	0.37 (0.02, 1.44)	0.6722
Birthweight (g)		52.91 (27.77, 78.04)	<0.0001	19.11 (−2.48, 40.70)	0.0832
Z-score		0.03 (−0.03, 0.08)	0.3333	0.03 (−0.03, 0.08)	0.3750
LBW (<2500 g)		0.60 (0.47, 0.77)	<0.0001	0.85 (0.55, 1.29)	0.4411
Very LBW (<1500 g)		0.19 (0.08, 0.47)	0.0003	0.57 (0.01, 1.11)	0.5997
Fetal macrosomia (≥4000 g)		1.20 (0.97, 1.48)	0.0982	1.22 (0.93, 1.61)	0.1474
SGA (<10th percentile)		0.86 (0.72, 1.03)	0.1090	0.83 (0.67, 1.03)	0.0922
Very SGA (<3rd percentile)		0.76 (0.54, 1.09)	0.1334	0.71 (0.44, 1.13)	0.1434
LGA (>90th percentile)		1.04 (0.86, 1.25)	0.7138	1.14 (0.91, 1.42)	0.2504
Very LGA (>97th percentile)		0.93 (0.65, 1.33)	0.6996	0.97 (0.63, 1.51)	0.9101

Analyses were adjusted for maternal age, paternal age, maternal BMI, basal FSH, anti-Müllerian hormone (AMH), type of infertility, prior gonadotropin cycle, ovarian stimulation protocols, dosage of gonadotropins, estrogen (E2) level on the hCG trigger day, number of oocytes retrieved, fertilization method, number and stage of embryos transferred, and gender of newborn; birthweight outcomes additionally adjusted for GA, preterm delivery (PTD), and very PTD.

LBW, low birthweight; LGA, large for gestational age; SGA, small for gestational age; OR, odds ratio; CI, confidence interval; Ref, reference group.

* Statistically significant, with *P *<* *0.05.

Furthermore, the incidence of PTD was significantly decreased with increasing EMT (adjusted odds ratio (OR): 0.55, 95% CI: 0.20 to 0.75; *P* = 0.0062). When the EMT was categorized into four groups, the odds of PTD were reduced by 45% with an EMT of 9.00–9.90 mm (adjusted OR: 0.55, 95% CI: 0.13 to 0.98; *P* = 0.0451), 58% with an EMT of 10.00–10.90 mm (adjusted OR: 0.42, 95% CI: 0.06 to 0.87; *P* = 0.0211) and 75% with an EMT >11.00 mm (adjusted OR: 0.25, 95% CI: 0.04 to 0.66; *P* = 0.0034) compared to the reference group (6.00–8.90 mm).

However, there was no significant difference in the rates of birthweight, LBW, very LBW, fetal macrosomia, very PTD, SGA, very SGA, LGA, or very LGA when the analyses were adjusted for confounding variables and birthweight outcomes were additionally adjusted for GA, PTD, and very PTD.

**Figure 2.**
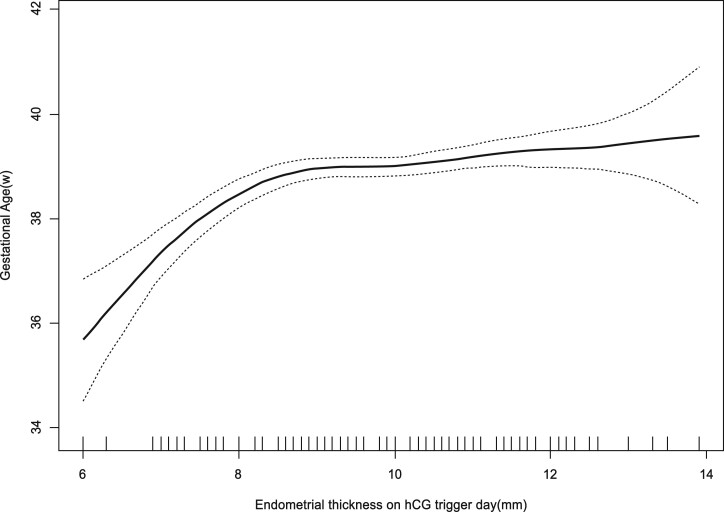
**The relationship between endometrium thickness on the hCG trigger day and adjusted mean gestational age.** Analyses were adjusted for maternal age, paternal age, maternal BMI, basal FSH, anti-Müllerian hormone (AMH), type of infertility, prior gonadotropin cycle, ovarian stimulation protocols, dosage of gonadotropins, estrogen (E2) level on the hCG trigger day, number of oocytes retrieved, fertilization method, number and stage of embryos transferred, and gender of newborn. GA: gestational age.

**Figure 3.**
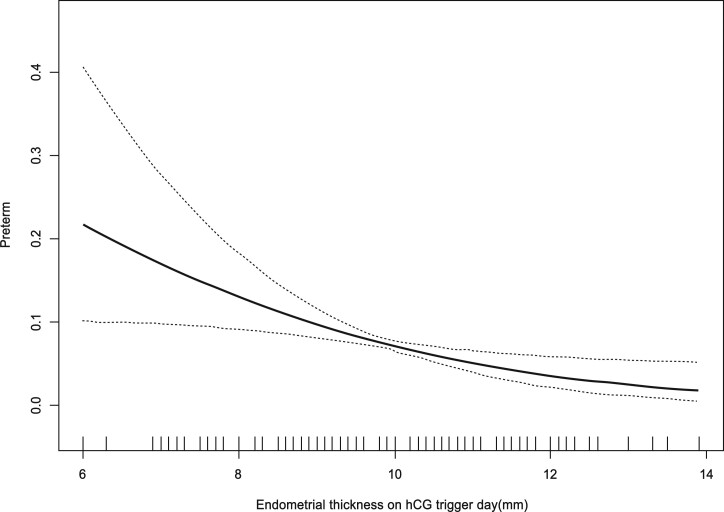
**The relationship between endometrium thickness on the hCG trigger day and adjusted preterm delivery incidence.** Analyses were adjusted for maternal age, paternal age, maternal BMI, basal FSH, anti-Müllerian hormone (AMH), type of infertility, prior gonadotropin cycle, ovarian stimulation protocols, dosage of gonadotropins, estrogen (E2) level on the hCG trigger day, number of oocytes retrieved, fertilization method, number and stage of embryos transferred, and gender of newborn.

The curves in Figs 2 and 3 show the relationship between the EMT and the adjusted mean GA (weeks) and the adjusted PTD incidence, respectively. Combined with the threshold effect of the EMT (mm) on GA outcomes by using piecewise linear regression displayed in Table 3, the analysis indicated that when EMT was ≤7.8 mm, GA increased significantly with increasing EMT (adjusted β: 1.94, 95% CI: 1.26 to 2.63; *P* < 0.0001), and when EMT was ≤7.6 mm, the incidence of PTD decreased obviously with increasing EMT (adjusted OR: 0.47, 95% CI: 0.03 to 0.99; *P* = 0.0107). This may indicate that 7.8 mm is the inflection point where the EMT affects the outcome of GA.

**Table 3:** Threshold effects of endometrial thickness on gestational age and the incidence of preterm delivery using piece-wise linear regression.

Characteristics	Crude β/OR	*P*-value	Adjust β/OR	*P*-value
	(95% CI)		(95% CI)	
Gestational age (weeks)				
≤7.8 mm	1.56 (1.04, 1.97)	0.0027	1.94 (1.26, 2.63)	<0.0001*
>7.8 mm	0.11 (0.01, 0.21)	0.0332	0.10 (−0.01, 0.20)	0.0640
Incidence of preterm delivery				
≤7.6 mm	0.63 (0.01, 0.99)	0.0302	0.47 (0.03, 0.99)	0.0107*
>7.6 mm	0.85 (0.15, 3.37)	0.2871	0.68 (0.32, 1.29)	0.1589

Analyses were adjusted for maternal age, paternal age, maternal BMI, basal FSH, anti-Müllerian hormone (AMH), type of infertility, prior gonadotropin cycle, ovarian stimulation protocols, dosage of gonadotropins, estrogen (E2) level on the hCG trigger day, number of oocytes retrieved, fertilization method, number and stage of embryos transferred, and gender of newborn.

OR, odds ratio; CI, confidence interval.

* Statistically significant, with *P *<* *0.05.

The univariate linear analysis shown in Table 4 revealed that six factors significantly influenced GA (weeks), including maternal age (unadjusted β: −0.03, 95% CI: −0.04 to −0.01; *P* = 0.0085), paternal age (unadjusted β: −0.02, 95% CI: −0.03 to −0.00; *P* = 0.0323), maternal BMI (unadjusted β: −0.03, 95% CI: −0.05 to −0.00; *P* = 0.0240), prior gonadotropin cycles (unadjusted β: −0.24, 95% CI: −0.47 to −0.01; *P* = 0.0397), EMT (mm) (unadjusted β: 0.07, 95% CI: 0.02 to 0.12; *P* = 0.0067), and gender of the newborn (unadjusted β: 0.29, 95% CI: 0.01 to 0.56; *P* = 0.0391).

**Table 4:** Univariate analysis of impact factors on gestational age (n = 2010).

Exposure	Values	Change in gestational age (weeks)	
	**mean** **±** **SD/n (%)**	β (95% CI)	*P*-value
Maternal age (year)	30.92 ± 3.99	−0.03 (−0.04, −0.01)	0.0085*
Paternal age (year)	32.45 ± 4.77	−0.02 (−0.03, −0.00)	0.0323*
Maternal BMI (kg/m^2^)	22.30 ± 3.15	−0.03 (−0.05, −0.00)	0.0240*
Basal FSH (IU/l)	7.64 ± 2.45	0.01 (−0.02, 0.04)	0.6667
AMH (ng/ml)	2.85 ± 2.24	0.03 (−0.00, 0.06)	0.0768
Type of infertility			
Primary	1019 (50.70%)	Ref	
Secondary	991 (49.30%)	0.14 (−0.13, 0.41)	0.3113
Infertility duration (year)	3.69 ± 2.71	−0.04 (−0.09, 0.01)	0.1149
Infertility cause			
Female	1304 (64.88%)	Ref	
Male	397 (19.75%)	−0.15 (−0.51, 0.21)	0.4146
Mixed	189 (9.40%)	0.23 (−0.30, 0.76)	0.3891
Unexplained	120 (5.97%)	−0.25 (−0.78, 0.29)	0.3663
Prior gonadotropin cycle(s)	1.22 ± 0.55	−0.24 (−0.47, −0.01)	0.0397*
Ovarian stimulation protocols			
GnRH-a long protocol	1275 (63.43%)	Ref	
Antagonist protocol	735 (36.57%)	−0.14 (−0.30, 0.03)	0.0982
Dosage of gonadotropins (100 IU)	22.89 ± 11.17	−0.00 (−0.01, 0.00)	0.4788
Stimulation duration (days)	11.57 ± 2.20	0.00 (−0.03, 0.04)	0.7997
E2 level on hCG day (100 pg/ml)	29.98 ± 12.71	0.00 (−0.00, 0.01)	0.3504
Number of oocytes retrieved	9.66 ± 3.83	0.01 (−0.03, 0.04)	0.6245
Fertilization method			
IVF	1331 (66.22%)	Ref	
ICSI	580 (28.86%)	0.12 (−0.19, 0.43)	0.4520
IVF + ICSI	99 (4.93%)	−0.10 (−0.76, 0.56)	0.7659
Stage embryo transferred			
D3	1673 (83.23%)	Ref	
D5	337 (16.77%)	0.09 (−0.30, 0.49)	0.6469
Number of embryos transferred			
1	550 (27.36%)	Ref	
2	1455 (72.39%)	−0.11 (−0.43, 0.20)	0.4786
3	5 (0.25%)	1.60 (−1.92, 5.12)	0.3740
Endometrial thickness (mm)	9.97 ± 1.54	0.07 (0.02, 0.12)	0.0067*
6.00–8.90 mm (n = 378)	378 (18.81%)	Ref	
9.00–9.90 mm (n = 444)	444 (22.09%)	0.22 (−0.01, 0.46)	0.0659
10.00–10.90 mm (n = 541)	541 (26.92%)	0.32 (0.09, 0.55)	0.0055*
>11.00 mm (n = 647)	647 (32.19%)	0.34 (0.12, 0.56)	0.0021*
Endometrial type			
A	30 (1.49%)	Ref	
A-B	107 (5.32%)	0.10 (−1.14, 1.34)	0.8739
B	620 (30.85%)	−0.09 (−1.17, 0.98)	0.8686
B-C	1109 (55.17%)	−0.28 (−1.34, 0.79)	0.6075
C	144 (7.16%)	−1.15 (−2.33, 0.02)	0.0555
Gender of newborn			
Male	1022 (50.85%)	Ref	
Female	988 (49.15%)	0.29 (0.01, 0.56)	0.0391*

AMH: anti-Müllerian hormone; E2: estrogen; GNRH-a: GNRH agonist; Ref, reference group; SD, standard deviation; CI, confidence interval.

* Statistically significant, with *P *<* *0.05.

In order to adjust confounding factors as much as possible, we have performed propensity score analysis. Since the exposure indicator, EMT (mm) is a continuous variable, we used the outcome indicator PTD for propensity score matching (PSM). According to the size of samples, matching is carried out at a ratio of 1:10, allowing a difference range of 0.05 in PSM. We constructed a regression model after matching propensity scores. The results, presented in Supplementary Table S1, indicated that the incidence of PTD decreased significantly with increasing EMT (adjusted OR: 0.59, 95% CI: 0.35 to 0.87; *P* = 0.0052), which was consistent with the results in Table 2.

## Discussion

The main objective of our current study, in which 2010 singleton births of non-PCOS infertile women under 42 years old were enrolled, was to evaluate the relationship between EMT on the hCG trigger day and neonatal outcomes after fresh ET. After adjusting for potential confounders, we found a strong negative correlation between EMT and the incidence of PTD, regardless of whether EMT was taken as a continuous or a categorical variable. In addition, the results of curve fitting and the threshold effect showed that the exact cutoff value was 7.8 mm. This may indicate that an EMT ≤7.8 mm is an independent factor for greater odds of PTD singletons born after fresh ET. To our knowledge, this is the first study in which the exact cutoff value of the impact of EMT on neonatal outcomes has been calculated through smooth curve fitting and threshold effect analysis using piecewise linear regression.

In recent years, increasing attention has been paid to the relationship between EMT and maternal and fetal safety, not just to pregnancy outcomes. Chung *et al.* (2006) first reported that suboptimal endometrial development with an EMT ≤10 mm was associated with a 2-fold increased risk of LBW compared with an EMT >12 mm in fresh IVF cycles. However, it is not clear whether this increased risk of LBW was adjusted for GA and PTD. The results of a recent study with a sample size of 5220 suggested that individuals with an EMT <8 mm had higher risks for both PTD (adjusted OR: 1.75, 95% CI: 1.30–2.34) and LBW (adjusted OR: 1.57, 95% CI: 1.09–2.26) than those with an EMT >8 mm (Hu *et al.*, 2021). Nevertheless, the birthweight outcome was flawed without ruling out GA and pregnancy complications. In our study, we also found that the birthweight increased (3123.48 ± 491.04 vs 3201.79 ± 556.65 vs 3191.90 ± 446.38 vs 3276.62 ± 495.47, *P* = 0.025) with endometrial thickening. However, after adjusting for confounding factors, especially including GA and PTD, the birthweight gain was not significant (*P* > 0.05). This result indicated that the change in birthweight was closely related to gestational weeks, and there was no independent relationship between birthweight and EMT. Huang *et al.* (2020) demonstrated in IUI cycles, that EMT was also not independently associated with LBW. Previous studies have shown that a strong negative correlation between the incidence of PTD and EMT can also be seen in PCOS patients (Huang *et al.*, 2021). However, to prevent any basis for adverse neonatal outcomes associated with abnormal glucose and lipid metabolism, we excluded patients with PCOS diagnosed via the Rotterdam criteria.

Different studies have generated different conclusions in terms of the cutoff value of EMT for the effect on neonatal outcomes. Chung *et al.* (2006) considered an EMT of 8 mm as the cutoff value (Hu *et al.*, 2021; Huang *et al.*, 2021), and some regarded the inflection point as 7.5 mm (He *et al.*, 2022) or 10 mm (Chung *et al.*, 2006). However, the cutoff values of EMT in these studies were almost based on empirical grouping. Our study is the first to use smooth curve fitting and threshold effect analysis to explore the exact inflection point. We found that when EMT was ≤7.8 mm, GA started to decline markedly with EMT thinning. This result indicated that the accurate cutoff value of the thin EMT that leads to an increase in the odds of PTD is 7.8 mm.

At present, the mechanism by which thin EMT affects neonatal outcomes is still being explored. Researchers in most previous studies have mentioned that this may be related to differences in oxygen tension. Early studies showed that after ovulation, the spiral artery contracts, resulting in a decrease in blood flow on the surface of the endometrium, thereby reducing the oxygen tension of the functional epithelium (Rossman and Bartelmez, 1957; Casper, 2011). This hypoxic stress in the villus space in the early pregnancy is the main prerequisite for normal embryogenesis and fetal development (Schoots *et al.*, 2018). However, when the functional layer is thin or absent, the oxygen concentration in the blood vessels of the basal endometrium will be considerably increased. This high concentration of reactive oxygen species may create a harmful uterine environment for the implants and placenta, ultimately leading to adverse growth of the placenta and fetus (Ribeiro *et al.*, 2018; Gupta *et al.*, 2009).

Another mechanism may be related to defects in spiral artery remodeling. Deep placental formation involves trophoblasts invading the medial third of the myometrium of the uterus and completely transforming the spiral arteries in this area (Brosens *et al.*, 2011). Low resistance of uteroplacental circulation is essential for the optimal development of the fetus (Brosens *et al.*, 2002). A thin endometrium is characterized by high uterine artery blood flow impedance due to vascular dysplasia (Miwa *et al.*, 2009). These abnormalities may change the remodeling of vascular spiral arteries and may affect the causes of abnormal placentation and adverse neonatal outcomes (Oron *et al.*, 2018). Maternal complications are closely related to abnormal neonatal outcomes. Abnormal formation of the placenta and its blood vessels may cause placental diseases in the mother, such as placenta previa, cesarean section, and HDP. Jing *et al.* (2019) reported that after adjusting for confounders, they found that a thicker EMT was associated with a decreased risk of placenta previa (adjusted OR: 0.798, 95% CI: 0.651–0.979; *P* = 0.031) and a decreased risk of cesarean section (adjusted OR: 0.926, 95% CI: 0.889–0.965; *P* < 0.001). The results of a large sample study that involved 9266 women who had singleton livebirths after fresh IVF/ICSI-ET cycles confirmed that a thin endometrium (≤8 mm) was associated with an increased risk of HDP, indicating that the thin endometrium itself is a risk factor for HDP (Liu *et al.*, 2021). The above-mentioned maternal complications may result in a decrease in the GA of newborns and increase in the incidence of PTD. In our study, women with maternal complications during pregnancy were excluded.

The main strength of this work is that this is the first study in which smooth curve fitting and threshold effect analysis, rather than just empirical grouping, have been used to explore the exact cutoff value of EMT. A second strength is that we made our best efforts to remove all confounding factors that could interfere with the results. We excluded patients with PCOS and patients with maternal complications during pregnancy, such as HDP, gestational diabetes, placenta previa, and placental abruption, which were likely to affect neonatal outcomes. In addition, to explore the independent effect of the EMT on neonatal outcomes, we also performed a regression analysis after PSM. The results of the PSM are consistent with the results of multivariable regression analysis in Table 2. This indicates that there is indeed a strong negative correlation between EMT and the incidence of PTD in fresh cycles.

However, the power of this study is limited owing to its retrospective design. A second limitation is that although we found a significant decrease in PTD as the EMT increased, in terms of GA, the magnitude of the differences was modest, possibly limiting the clinical relevance of the findings. In addition, it has still not been ruled out that the internal factors, such as previous medication, nutrition intake, and living habits, may cause bias in the neonatal outcomes.

## Conclusion

In conclusion, in singletons born of non-PCOS patients after fresh ET, EMT on the hCG trigger day is negatively correlated with PTD. Our data also demonstrated that an EMT ≤7.8 mm was an independent predictor for greater odds of PTD after fresh ET. Therefore, we suggest that, to reduce the incidence of PTD, women with thin EMT after conception via fresh IVF-ET cycles should receive more attention from obstetricians and pediatricians.

## Supplementary Material

hoad028_Supplementary_Data

## Data Availability

The data underlying this article will be shared upon reasonable request to the corresponding author.
