# Supplementary material for: The thicker the endometrium, the better the neonatal outcomes?
Source: Hum Reprod Open. 2023 Jul 13;2023(3):hoad028. doi: 10.1093/hropen/hoad028 (PMC10363027; doi:10.1093/hropen/hoad028)
Supplement: hoad028_Supplementary_Data [file hoad028_supplementary_data.docx]

Supplementary Table S1: Association of endometrial thickness and preterm delivery after Propensity-Score Matching.

| **Characteristics** | **Effect Estimate, Odds Ratio (95% CI)** | | | | | |
| --- | --- | --- | --- | --- | --- | --- |
|  | **Non-adjusted** | ***P* value** | **Matched on Age, AMH, Gn cycles and total Gn dose** | ***P* value** | **Propensity Score Adjusted** | ***P* value** |
| Preterm (32-36w) | 0.77 (0.63, 0.95) | 0.0129 | 0.67 (0.39, 0.93) | 0.0077^*^ | 0.59 (0.35, 0.87) | 0.0052^*^ |

Gn, Gonadotropin; CI, confifidence interval.

*Statistically significant, with *P* < 0.05.
